# Contraception matters: indicators of poor usage of contraception in sexually active women attending family planning clinics in Victoria, Australia

**DOI:** 10.1186/1471-2458-12-1108

**Published:** 2012-12-23

**Authors:** Jason Ong, Meredith Temple-Smith, William CW Wong, Kathleen McNamee, Christopher Fairley

**Affiliations:** 1 Department of General Practice, University of Melbourne, Melbourne, VIC, Australia; 2 General Practice and Primary Health Care Academic Centre, University of Melbourne, Melbourne, VIC, Australia; 3 Department of Family Medicine and Primary Care, University of Hong Kong, Pokfulam, Hong Kong; 4 Department of Obstetrics and Gynaecology, Family Planning Victoria. Monash Medical Centre, Clayton, VIC, Australia; 5 Melbourne Sexual Health Centre. School of Population Health, University of Melbourne, Melbourne, VIC, Australia

**Keywords:** Contraception, Family Planning, Women’s health, Unintended pregnancy, Unplanned pregnancy

## Abstract

**Background:**

Unintended pregnancy (mistimed or unwanted) remains an important health issue for women. The purpose of this study was to determine the prevalence of and factors associated with risk of unintended pregnancy in a sample of Victorian women attending family planning clinics.

**Methods:**

This cross-sectional survey of three Family Planning Victoria Clinics from April to July 2011 recruited *w*omen aged 16-50 years with a male sexual partner in the last 3 months, and not intending to conceive. The questionnaire asked about contraceptive behaviours and important factors that influence contraception use (identified from a systematic literature review). Univariate analysis was calculated for the variables of interest for associations with contraceptive use. An overall multivariate model for being at risk for unintended pregnancy (due to inconsistent or ineffective contraceptive use or non-use) was calculated through backward elimination with statistical significance set at <0.05.

**Results:**

1006 surveys were analyzed with 96% of women reporting contraception use in the last 3 months. 37% of women were at risk for unintended pregnancy due to imperfect use (61% inconsistent users; 31% ineffective methods) or never using contraception (8%). On multivariate analysis, women at risk for unintended pregnancy compared with women not at risk were <25 years old (OR 1.8, 95% CI 1.2-2.7); had no university/postgraduate degree (OR 1.7, 95% CI 1.2-2.4); and had >1 partner in the last 3 months (OR 3.2, 95% CI 2.3-4.6). These women were dissatisfied with current contraception (OR 2.5, 95% 1.8-3.5); felt “vulnerable” to pregnancy (OR 2.1, 95% CI 1.6-3.0); were not confident in contraceptive knowledge (OR 2.6, 95% CI 1.5-4.8); were unable to stop to use contraception when aroused (OR 2.1, 95% CI 1.5-2.9) but were comfortable in speaking to a doctor about contraception (OR 2.3, 95% CI 1.1-4.1).

**Conclusion:**

Despite reported high contraceptive usage, nearly 40% of women were at risk for unintended pregnancy primarily due to inconsistent contraceptive use and use of ineffective contraception. Strategies for improving consistency of effective contraception use or greater emphasis on long-acting contraception may be needed for certain subpopulations at higher risk for unintended pregnancy.

## Background

Unintended pregnancy, defined as *mistimed* (occurring earlier than desired) or *unwanted* (occurring when no more children are desired) is estimated to account for 80 million of the 210 million pregnancies that occur worldwide each year [1]. Whilst there is no accurate data on the intendedness of births in Australia, a recent cross-sectional survey of a nationally representative sample of Australian women of reproductive age found 51% had experienced an unintended pregnancy in their lifetime [2]. Weisberg’s study of 811 Australian women (as a part of the Australian Longitudinal Study of Women’s Health) found that 32% of first pregnancies were unplanned and 29% were unwanted [3].

In 2008, there was a call to action by key sexual and reproductive health organizations in Australia, which highlighted a significantly higher teenage birth abortion rates compared to other developed countries. Also identified were unacceptably high levels of reproductive ill health defined by inconsistent access to, and use of, a full range of available contraceptive methods [4].

Access to, and utilization of, pregnancy planning services including proper use of safe and effective contraception, plays an important role in combating the problem of unintended pregnancy. Key players in the prevention of unintended pregnancies are doctors, particularly General Practitioners, who see 85% of the population yearly [5]. A recent meta-analysis evaluating 26 randomized controlled pregnancy prevention programs (school based, family planning, community based programs) showed that none had a significant impact in improving the use of contraception or in reducing the number of pregnancies among adolescents [6]. However there is evidence that brief counselling interventions by doctors are effective in modifying health behaviours, especially in adolescents [7].

Whilst it seems intuitive to then encourage *all* doctors to ask *every* woman about their contraceptive and sexual behaviours, in reality this is not feasible for time-limited doctors with multiple demands on their attention. There is a need for population and setting specific information that better identifies women at greater vulnerability of unintended pregnancy that can be used by *local* health professionals to benefit the sexual health of women.

## Methods

Ethical approval was obtained from the University of Melbourne Human Ethics Advisory Group (ID 1135498) and Family Planning Victoria Human Research Ethics Committee (S11030412000).

### Selection and description of participants

Women aged 16-50 years attending Family Planning Victoria’s three clinics during the period April to July 2011 were recruited. These women were sexually active with at least one male partner in the last 3 months, but not trying to conceive. The triage nurse invited all eligible women to complete the anonymous but numbered questionnaire prior to seeing the doctor. Following receipt of the survey, participants placed their completed or uncompleted survey in a secure box thus ensuring that a record was obtained of the number of non-responders.

Recruitment had to be free of coercion. At triage, the nurse would stress to the woman that the questionnaire was entirely anonymous, voluntary and would not impact on her consultation. The patient information sheet the woman received also stressed the anonymous and voluntary nature of the study. No reimbursements or payment were given to women involved in the study. The women had complete autonomy during the whole recruitment process as they had the right to not complete or hand in the survey, without the knowledge of the triage nurse.

Family Planning Victoria was chosen as the site of the survey as the majority of its clients matched the intended target population and fulfilled the inclusion criteria, allowing rapid uptake of the questionnaire. Family Planning Victoria is an independent, not-for-profit organization that is partially funded by the Victorian government. It provides clinical care in sexual and reproductive health. The Action Centres are drop-in centres in Melbourne central business district and Hoppers Crossing (outer metropolitan), specifically catering for people under 25 years old. The Box Hill Centre (inner suburban) caters for all age groups with both an appointment system and drop-in services. A small administration fee is charged yearly which gives unlimited access to the clinics where consultations are low cost or free.

### Questionnaire

The questionnaire comprising 34 items covered demographics, frequency and type of contraceptive use. Through a literature review of factors that may affect the use of contraception, domains were developed to capture the breadth of potential factors [8]. Questions from these domains were compiled into the questionnaire which can be made available on request to the corresponding author. The questionnaire was initially pilot tested with 10 sexually active females from Victoria (mean age 23 years old) for comprehensibility and content validity.

Domains of factors that may affect contraception use:

• Demographics (age, suburb of residence, country of birth, year of arrival in Australia, main language at home, possession of health care card or private health insurance)

• Social norms (comfort in discussing contraception with the doctor/parents/partner/friends, support of contraceptive use from parents/partner/friends, partner’s refusal to use contraception)

• Social circumstances (annual income, SEIFA score^*^, access to contraception, embarrassment about speaking to the doctor about contraception, contraception too expensive, forgetting to use contraception)

• Information (confidence in knowledge of pregnancy prevention, highest education level completed)

• Experiences (contraceptive side effects, previous pregnancy, previous unintended pregnancy, outcome of pregnancy)

• Beliefs (satisfaction with current contraception, vulnerability to becoming pregnant, concern about the effects of hormones, contraception too expensive, contraceptive side effects, inconvenience in using contraception, access to contraception when needed, embarrassment in discussing or buying contraception)

• Cue to action (health professional discussing contraception in the last 12 months, number of sexual partners, frequency of sex)

• Self concept (contraceptive self-efficacy)

• Motivation (importance of preventing pregnancy at this stage of their life)

* SEIFA stands for Socio-economic Indexes for Areas. A SEIFA score (Index of Relative Socio-economic Advantage and Disadvantage (IRSAD)) is a summary measure of a number of variables that represent different aspects of relative socio-economic disadvantage and/or advantage in a geographic area. A low score indicates relatively greater disadvantage and a lack of advantage in general. A high score indicates a relative lack of disadvantage and greater advantage in general.

The survey took approximately seven minutes to complete. Women at risk for *unintended* pregnancy were defined as sexually active women who stated it was ‘very important’ or ‘important’ to avoid pregnancy at this stage in their life and yet were using contraception inconsistently or using an ineffective contraception. Inconsistent contraception use was determined from the question ‘of the times you had sex in the last 3 months was contraception used either by you or the other person or both? (Never 0%, Not usually 1-50%, Sometimes 50-75%, Most of the time 76-99%, Always 100%).’ Those who did not tick ‘always’ were defined as inconsistent users. Ineffective contraception was defined as any method with a failure rate of more than 10% in the first year of usage (i.e. sole use of female condom, withdrawal, spermicide, rhythm).

### Statistics

The data was entered into the statistical package MINITAB (v. 16.1.0). The statistical modeling focused on the use of various contraceptives and being at risk of unintended pregnancy as the major binary outcomes of interest. Univariate logistic regression was performed for the variables in each of the eight domains and is presented in Table 1. The explanatory variables in each of these domains were then used in an overall multivariate model for being at risk for unintended pregnancy, using backward elimination. Statistical significance was set at <0.05. The Hosmer-Lemeshow test, a statistical test for goodness of fit for logistic regression model was performed. Some 2-way interactions were also considered for some major variables (e.g. age and level of education, confidence of contraceptive knowledge and level of education) but were not added to the model as it did not add any new information to the final multivariate model.

**Table 1:** Univariate analysis of factors associated with women at risk for unintended pregnancy

**Explanatory variable**	**At risk for unintended pregnancy**		**Not at risk for unintended pregnancy**		**Odds ratio**		
	***n***	**%**	***n***	**%**	**Estimate**	**Confidence interval**	***p *value**
***DEMOGRAPHICS DOMAIN***							
***Age***							
**<25 years old (vs. >25 years old)**	**297**	**80**	**380**	**63**	**2.4**	**1.8, 3.3**	**<0.0001**
***Birthplace***							
**Born in Australia (vs. Born overseas)**	**286**	**77**	**435**	**72**	**1.3**	**1.0, 1.8**	**0.056**
*Time in Australia*							
<5 years (vs. >5 years)	39	11	78	13	0.8	0.5, 1.2	0.286
*Language at home*							
English (vs. non-English)	331	90	551	91	0.8	0.5, 1.3	0.407
***SOCIAL NORMS DOMAIN***							
*Discussion of contraception with doctor*							
Comfortable (vs. not comfortable)	336	93	561	94	0.9	0.5, 1.5	0.614
*Discussion of contraception with parents*							
Comfortable (vs. not comfortable)	105	38	165	45	0.7	0.5, 1.0	0.066
***Discussion of contraception with partner***							
**Comfortable (vs. not comfortable)**	**292**	**89**	**528**	**95**	**0.4**	**0.2, 0.7**	**<0.0001**
*Discussion of contraception with friends*							
Comfortable (vs. not comfortable)	311	92	500	91	1.2	0.7, 1.9	0.557
*Contraceptive support from parents*							
Supportive (vs. not supportive)	193	69	273	72	0.9	0.6, 1.2	0.379
***Contraceptive support from partner***							
**Supportive (vs. not supportive)**	**295**	**85**	**556**	**94**	**0.3**	**0.2, 0.5**	**<0.0001**
***Contraceptive support from friends***							
**Supportive (vs. not supportive)**	**335**	**95**	**531**	**91**	**1.8**	**1.1, 3.1**	**0.029**
***SOCIAL CIRCUMSTANCES DOMAIN***							
*SEIFA (decile group)*						χ^2^_2_ = 1.8, p =0.404	
1-5	33	9	69	11	*reference*	-	
6-8	82	22	140	23	1.2	0.8, 2.0	0.424
9-10	253	69	394	65	1.3	0.9, 2.1	0.193
***Health Care Card***							
**Yes (vs. no)**	**150**	**41**	**192**	**32**	**1.5**	**1.1, 2.0**	**0.004**
*Private Health Insurance*							
Yes (vs. no)	170	46	293	49	0.9	0.7, 1.2	0.508
***Annual Household income***							
**>$60,000 (vs. <$60,000)**	**99**	**34**	**215**	**43**	**0.7**	**0.5, 0.9**	**0.017**
***INFORMATION DOMAIN***							
***Highest Education completed***							
**University/PostGraduate (vs. no University/Post graduate)**	**91**	**25**	**244**	**40**	**0.49**	**0.4, 0.7**	**<0.0001**
***Confident in knowledge of pregnancy prevention***							
**Yes (vs. No)**	**314**	**85**	**575**	**95**	**0.32**	**0.2, 0.5**	**<0.0001**
***PAST EXPERIENCES DOMAIN***							
**Ever pregnant**							
**Yes (vs. No)**	***73***	**20**	***173***	**29**	**0.6**	**0.5, 0.9**	***0.003***
Ever unintended pregnancy							
Yes (vs. No)	*59*	16	*117*	19	0.8	0.6, 1.1	*0.212*
**Ever full term**							
**Yes (vs. No)**	***17***	**5**	***46***	**8**	**0.6**	**0.3, 1.1**	***0.072***
Ever miscarriage							
Yes (vs. No)	*14*	4	*21*	4	1.1	0.6, 2.2	*0.778*
Ever abortion							
Yes (vs. No)	*38*	10	*80*	13	0.8	0.5, 1.2	*0.195*
*BELIEFS DOMAIN*							
**Feel vulnerable to pregnancy**							
**Yes (vs. No)**	***193***	**50**	***187***	**32**	**2.5**	**1.9, 3.2**	***<0.0001***
**Satisfied with contraception**							
**Yes (vs. No)**	***154***	**42**	***440***	**73**	**0.3**	**0.2, 0.4**	***<0.0001***
***CUE TO ACTION DOMAIN***							
Health professional has spoken about contraception in last 12 months							
Yes (vs. No)	*258*	74	*450*	76	0.9	0.6, 1.2	*0.319*
**Used >1 contraception in last 3 months**							
**Yes (vs. No)**	***99***	**27**	***231***	**38**	**0.6**	**0.5, 0.8**	***<0.0001***
**>1 partner in the last 3 months**							
**Yes (vs. No)**	***153***	**43**	***114***	**19**	**3.1**	**2.3, 4.2**	***<0.0001***
**Frequency of sex in the last 3 months**							
**2-4 times/week or daily (vs. 2-4 times/month or less)**	***153***	**43**	***114***	**19**	**3.1**	**2.3, 4.2**	***<0.0001***
***CONTRACEPTIVE SELF EFFICACY DOMAIN***							
**Plan ahead to have some form of contraception**							
**Yes (vs. no)**	***318***	**87**	***571***	**96**	**0.3**	**0.2, 0.5**	***<0.0001***
**Stop to use contraception once aroused**							
**Yes (vs. no)**	***219***	**60**	***476***	**80**	**0.4**	**0.3, 0.5**	***<0.0001***
**Resist sex if partner did not want to use contraception**							
**Yes (vs. no)**	***179***	**49**	***428***	**72**	**0.4**	**0.3, 0.5**	***<0.0001***

*Texts are bolded if p value <0.1.*

## Results

Of the 2184 women who were eligible during the study period, 1109 women were approached and 1024 were enrolled. Eighteen surveys were excluded because they had >50% of questions unanswered or were ineligible because of age. Thus, 1006 surveys were analyzed.

### Background of the study population

Women in this study averaged 24.3 years of age (SD = 8.0). The study sample is compared with the Victorian population in the Table 2. Notably, the study sample was a predominantly younger group of women with the majority born in Australia and speaking English at home. It is also important to note that compared to the patient population that attends Australian General Practice, this study population reported 69% of women less than 25 years old compared with 21% of GP patient encounters [9]. Furthermore only 3% of GP patient encounters are classified as ‘family planning’ related [9]. However, in terms of absolute numbers, more women would go to a General Practitioner for their contraceptive needs than to a family planning service.

**Table 2:** Demographics of study population vs. Victorian female population

**Variable**	**At risk for unintended pregnancy % (n)**	**Not at risk for unintended pregnancy % (n)**	**Total sample %**	**Victorian female population 2006 census **[10]**%**
*Age group (years)*				
16-19	43 (160)	26 (160)	32	7
20-24	37 (137)	36 (220)	36	7
25-29	9 (33)	14 (86)	12	7
30-34	3 (12)	7 (42)	6	7
35-39	2 (8)	8 (48)	6	8
40-50	5 (20)	9 (52)	8	15
*Highest education level completed*				
University or Postgraduate	25 (91)	40 (244)	34	32
*Country of Birth*				
Australia	77 (286)	72 (435)	73	70
*Language spoken at home*				
English	90 (331)	91 (551)	90	75
*Medical Insurance*				
Health care card	41 (150)	32 (192)	35	
Private insurance	46 (170)	49 (293)	47	*
*Household Income ($)*				
≥60,000	34 (99)	43 (215)	32	*
*SEIFA score*				
1-5	9 (33)	11 (69)	10	*
6-8	22 (82)	23 (140)	23	
9-10	69 (253)	65 (394)	66	

* Data not available from Census.

96% of women reported use of some forms of contraception in the last 3 months of which the most popular methods were male condoms (67%), oral contraceptive pill (49%) and withdrawal (32%) (Table 3). The majority of women (89%) reported confidence in their knowledge of how to prevent pregnancy and 87% of women stated it was important to avoid pregnancy at this stage in their life.

**Table 3:** Contraception use in the last 3 months (ever used and sole users)

	**Used in last 3 months**		**Sole users**	
**Contraception**	***n***	**%**	***n(sole users in last 3 months)/n(all users in last 3 months)***	**%**
Male condoms	672	67	227/672	34
Oral contraceptive pill	488	49	146/488	30
Withdrawal	317	32	128/317	40
Emergency Contraception	131	13	4/131	3
Implanon NXT®	119	12	55/119	46
Rhythm	62	6	28/62	45
Intrauterine device	57	6	35/57	61
Depot medroxyprogesterone acetate	24	2	8/24	33
NuvaRing®	20	2	5/20	25
Female Condoms	10	1	1/10	10
Diaphragm	9	1	5/9	56
Vasectomy	8	1	2/8	25
Tubal ligation	6	1	5/6	83
*Total*	*1006*			

### Women at risk for unintended pregnancy

370 women (37%) of the sample population were at risk for *unintended* pregnancy: 61% due to inconsistent contraception use, 31% due to sole use of an “ineffective” contraception (98 solely using withdrawal, 18 solely using rhythm method) and 8% due to non use of contraception.

The two most common reasons for not using contraception consistently were “I did not have access to contraception when I needed it” (39%) and “I just forgot” (35%) (Table 4). An important analysis was to identify the consistency of use of women who were solely reliant on these methods (Table 5). Of note, 24% of *sole* condom users, 66% of *sole* oral contraceptive pill users and 80% of *sole* withdrawal users reported using these methods inconsistently.

**Table 4:** Reasons for inconsistent contraception use in the last 3 months

	***n***	**%**
No access	143	39
Forgot	130	35
Inconvenient	54	15
Side effects	39	11
Partner refused	34	9
Concerned about “hormones”	32	9
Too expensive	29	8
Didn’t feel need	23	6
Feels better without contraception	13	4
Too embarrassed to buy	13	4
Too embarrassed to talk to doctor	11	3
*Other:* Alcohol, Lazy, Spur of moment, Condom broke	13	4
	*total =370*	

**Table 5:** Inconsistent use by sole users of contraception in the last 3 months

**Contraception**	**Inconsistent users**	**Total sole users**	**(Inconsistent users)/(Total sole users) %**
Male condoms	55	227	24
OCP	96	146	66
Withdrawal	102	128	80
Rhythm	25	28	89
NuvaRing®	0	0	0
Diaphragm	3	5	60
Female condom	1	1	100

Univariate analysis is summarised in Table 1 with the statistically significant factors (p < 0.1) in bold. On multivariate analysis (Table 6), after adjusting for all other risk factors (including income, education and age), women at risk for unintended pregnancy compared with women not at risk were less than 25 years old, had no university or postgraduate degree and had more than 1 partner in the last 3 months. They had attitudes of dissatisfaction with current contraception, felt “vulnerable” to pregnancy, were not confident in contraceptive knowledge, were unable to stop to use contraception when aroused but comfortable in speaking to a doctor about contraception.

**Table 6:** Multivariate logistic regression of factors associated with women at risk for unintended pregnancy

	Adjusted odds ratio*		
**Explanatory variable**	**Estimate**	**Confidence interval**	***p *value**
Age <25 years old	1.8	1.2, 2.7	0.003
Not completed University or Postgraduate	1.7	1.2, 2.4	0.005
More than 1 partner	3.2	2.3, 4.6	<0.0001
Not using more than 1 contraception	1.2	1.1, 1.3	<0.0001
Dissatisfied with contraception	2.5	1.8, 3.5	<0.0001
Not confident in contraceptive knowledge	2.6	1.5, 4.8	0.001
Cannot stop herself to use contraception	2.1	1.5, 2.9	<0.0001
Feels vulnerable to pregnancy	2.1	1.6, 3.0	<0.0001
Comfortable with discussing contraception with doctor	2.3	1.1, 4.1	0.024
		*Hosmer Lemeshow test, x*^*2*^*(8) =8.906, p = 0.350*	

*Adjusted for variables in bold in Table 1.

## Discussion

### Women at risk for unintended pregnancy – it’s more common than we think

Despite more than 95% of women reporting current contraceptive use and nearly 90% of these women stating it was important to avoid pregnancy at this stage in their life, 37% remained at risk for unintended pregnancy. The Australian Study of Health and Relationships (ASHR) reported 5% of women being at risk for pregnancy by not using contraception all the time [11]. This low number in contrast to our 37% at risk for unintended pregnancy may be due to several factors. The study population was a younger cohort, a population that could inherently be more at risk for unintended pregnancy [12]. ASHR did not investigate consistency of contraceptive use and this may lead to an underestimation of the women at risk for unintended pregnancy by assuming perfect use. Alternatively there may be an actual increase in the number of women over the last 10 years putting themselves at risk for unintended pregnancy.

### Inconsistent contraceptive use – a major cause of unintended pregnancy

61% of women were at risk for unintended pregnancy due to inconsistent contraception use. It must be noted that this study may be underestimating the proportion of women at risk for unintended pregnancy as it has been shown that reported inconsistent pill use is much less than electronically recorded missed pills [13]. Distinguishing inconsistent users is important as prospective studies demonstrate that those who use contraception inconsistently at baseline were likely to be inconsistent users 6 months later [14]. A study of 1,511 couples from Perth found that the incidence of unintended pregnancy was four times higher in couples with inconsistent contraception use [15]. In reducing rates of unintended pregnancy, this study provides compelling evidence of the greater need for focusing strategies on improving *consistency* of contraceptive use or the need to advocate for non-user dependent methods, the long acting reversible methods of contraception.

The top two reasons for not using contraception consistently were “I did not have access to contraception when I needed it” and “I just forgot”. Forgetting to use a contraception that required daily vigilance or is coitally dependent is not a surprise. Forgetting to take the pill remains a common complaint of women using the oral contraceptive pill [11]. “Access” is more than just increasing the ease of obtaining contraception. Despite providing free access to contraception, the UK still has one of the highest rates of teen pregnancies in the world [16]. Given that the study population was already “accessing” a family planning service, this reported “lack of access” may reflect other variables - embarrassment in purchasing or discussing contraception; fear of carrying of condoms being seen as a premeditation of sex; inability to anticipate sex especially in the context of alcohol [17]; fear of lack of confidentiality [18]; transport problems, difficulties of getting time off work/school, taking too long to get an appointment or even waiting times in clinics [19]. “Accessability to contraception” certainly was seen to be an issue in adolescent populations [19] but it still remains unclear why this is so. These are questions that need further exploration.

### Factors affecting contraceptive use

#### Age

It is clear from the literature that the highest proportion of unintended pregnancies in each age group occurs in younger women and women more than 40 years old [12]. Due to the skew towards the younger cohort, our study was not powered to detect a difference for women aged more than 40 years old. The literature suggests that for those less than 25 years old, this increased vulnerability may be due to higher fertility, shorter intervals between sex [20], having multiple partners [21], and/or less capacity to anticipate sex (especially in the context of alcohol) [17].

#### Educational attainment

Other studies have also demonstrated an association between educational attainment and contraceptive use [22,23]. One may speculate that less educated women may be less likely to have higher educational and career aspirations and less understanding of health and thus, less motivation to use contraception. Not all the studies demonstrated this effect though. A large Australian study of 9,134 women showed no associations between overall contraceptive use and education [11], although a difference in contraceptive choice according to educational attainment was found. The oral contraceptive pill was more common in those who had post-secondary education vs. less than secondary; whilst tubal ligation/hysterectomy were more common in those with less than secondary education compared to post-secondary education [11].

#### Number of sexual partners

Reporting more than 1 partner in the last 3 months as a factor for unintended pregnancy is consistent with other international studies [24] and may be due to several factors. Firstly, having the same partner for longer periods of time has been shown to be associated with consistent contraceptive use [25]. Secondly, the “nature” of the relationship also seemed to correlate with likelihood of using contraception. For instance, if a woman was in a more romantic, caring close relationship, the couples has a higher likelihood of using contraception [26] than those in casual and uncommitted relationships. There was also evidence that couples who reported intimate reasons for having sex had significantly increased odds of discussing contraception before first coitus [27]. Thirdly, women who are in more stable relationships may move from more sporadic barrier methods to more effective hormonal methods [28].

#### Attitudes towards contraception

The multivariate analysis showed that women at risk of unintended pregnancy, compared to those not at risk for unintended pregnancy, felt less satisfied with their current contraceptive method, less confident in their knowledge of contraception, felt more vulnerable to pregnancy and felt that they ‘could not stop themselves from using contraception when aroused’ (reflecting poorer contraceptive self-efficacy [29]). These are all important gateways that may facilitate the discussion of contraception by the doctor. Interestingly, we found that they actually reported being more comfortable in speaking to their doctor about contraception. This may further reflect their readiness to speak about contraception but may demonstrate the failure of previous health professionals in addressing this need. Our study was not able to determine if this was the case. This would be an area for further research. What is already known is that a majority of adolescents want to discuss about how to prevent pregnancy and sexually transmitted infections [30]. However whilst 50% of teens attending their doctor thought that pregnancy should be discussed, only 21% of the visits was it addressed by the doctor [31].

Although doctors have limited time with patients, they have a unique opportunity to influence contraceptive behaviours in a way that other resources, such as school systems, cannot. They can capitalize on their confidential one-to-one consultation to probe into sexual and contraceptive behaviours, expectations regarding contraception and pregnancy desires when counselling patients on contraceptive use and other health-promoting behaviours.

#### Dual contraception use

That women at risk for unintended pregnancy had lesser odds of using more than one contraception was an important finding, as these women were not just at risk for unintended pregnancy but may also be at risk for sexually transmitted infections. This was consistent with the only other comprehensive Australian study on dual contraception use, which showed only 2-46% of sexually active women also used a condom with their current contraceptive [32].

### Clinical implications for primary care providers

1) The report of current contraceptive use does not equate to protection against unintended pregnancy. Primary care providers should ask about the *type* and *consistency* of contraceptive use in women attending their practice.

2) Inconsistent contraceptive use plays a major role in putting women at risk for unintended pregnancy. Consider options of long-acting reversible contraceptives for those more likely at risk e.g. Implanon NXT®, intrauterine device, depot medroxyprogesterone acetate (DMPA).

### Limitations

The main limitations pertain to the setting of the survey and the survey itself. Women attending Family Planning Victoria are not representative of the Australian population nor the general practice population. Thus, the findings are restricted to the population of women attending reproductive health services. Further studies from general practice settings, hospital outpatients of Obstetrics/Gynaecology and community settings are also needed to provide a broader understanding of how women in these other settings are using contraception.

As the survey was a self report of contraceptive use, there may be a risk of recall bias and reporting bias. Single item measures for evaluating some complex factors (attitudes of partner/parents/friends, etc), may not be sufficiently sensitive or reliable to measure the intended predictor. The survey only assessed reported consistency of use and not how well the contraceptive method was used. For those reporting inconsistent use, there was no scope to determine if any sexual activity occurred during these periods of increased pregnancy risk. Finally, as the survey was a cross sectional design, the list of factors derived from the multivariate analysis must not be used to forecast future use of contraception in these women. There is a need for a longitudinal study to test these risk factors as true markers for the risk of unintended pregnancy.

## Conclusions

Women attending family planning clinics remain at high risk for unintended pregnancy due to inconsistent contraceptive use and use of less effective contraception. Within this population, there are also subpopulations that remain at higher risk for unintended pregnancy i.e. less than 25 years old, no university or postgraduate degree, more than 1 partner in the last 3 months, dissatisfaction with current contraception, feeling vulnerable to pregnancy, not confident in contraceptive knowledge, and unable to stop to use contraception when aroused. Family Planning services should ensure that all clients but especially those with characteristics associated with a greater risk for unintended pregnancy are asked about the *type* and *consistency* of use of contraception.

This study also demonstrated how a short questionnaire covering multiple domains may capture important information on contraceptive use and factors affecting its use. This may be replicated in various settings of interest to better identify women at greater risk for unintended pregnancy.

## Competing interests

The authors declare that they have no competing interests.

## Authors’ contributions

JO, MTS, KM and WW designed the study. KM contributed data. All authors analyzed the data. JO drafted the manuscript and all authors revised and approved the final version.

## Pre-publication history

The pre-publication history for this paper can be accessed here:

http://www.biomedcentral.com/1471-2458/12/1108/prepub
